# A multi-criteria evaluation system for arable land resource assessment

**DOI:** 10.1007/s10661-019-8023-x

**Published:** 2020-01-02

**Authors:** Feipeng Li, Wei Liu, Zhibo Lu, Lingchen Mao, Yihua Xiao

**Affiliations:** 1 School of Environment and Architecture, University of Shanghai for Science and Technology, Shanghai, 200093 China; 2 College of Environmental Science and Engineering, Tongji University, Shanghai, 200092 China; 3 Department of Biological and Environmental Science, University of Jyväskylä, 40014 Jyväskylä, Finland; 4 School of Environmental & Municipal Engineering, Qingdao University of Technology, Qingdao, 266033 China

**Keywords:** Multi-criteria evaluation system, Arable land resource, Integrated fertility index (IFI), Soil cleanliness index, Heavy metals, Comprehensive soil evaluation score

## Abstract

This study proposed a multi-criteria evaluation system for arable land resources by combining the soil integrated fertility index (IFI) with a soil cleanliness index (based on heavy metals and metalloid content). A total of 16 typical arable land units in Chongming District, China, were evaluated using the proposed evaluation system based on 104 collected soil samples in 16 towns. The comprehensive soil evaluation scores of arable lands in 16 towns were in the range of 90.7 to 99.2 with a mean of 96.2, indicating that the arable land in all 16 towns was at the level of excellent (≥ 90.0). Lower cleanliness indices had a significant impact on the final evaluation score. In comparison with single-index evaluation systems (i.e., the IFI or soil cleanliness index), the proposed multi-criteria system better reflects the quality of the soil. In the practice of arable land requisition and subsidy policy, the proposed multi-criteria evaluation system not only encourages farmers to preserve arable lands during farming but also helps agricultural authorities make effective and reliable management decisions.

**Electronic supplementary material:**

The online version of this article (10.1007/s10661-019-8023-x) contains supplementary material, which is available to authorized users.

## Introduction

Arable land is the basis of agricultural production, and its quality is essential for crop security and ecological sustainability (Stenberg 1999). It represents a key component in the synchronization of urban and rural development. Rapid economic development and industrialization degrade the arable land in China (Hu et al. 2016; Zhao et al. 2014). The joint report on the current status of soil contamination in China, issued by the Ministry of Environmental Protection and the Ministry of Land and Resources of the People’s Republic of China in 2014, revealed that more than 19.4% of agricultural soils have been contaminated according to the soil environmental quality limits (MEP 2014; National Environmental Protection Bureau 1995). During the last two decades, a number of studies have shown that heavy metal pollution in soils has been widespread in China (Chen et al. 1999; Hu et al. 2016; Khan et al. 2008; Teng et al. 2010; Zheng et al. 2016). According to the State Environment Protect Agency (SEPA) of China (2006), it is estimated that 12 million tons of grain is polluted by heavy metals every year and a total of 10 million ha of arable land in China has been polluted by heavy metals such as chromium (Cr), zinc (Zn), copper (Cu), and Zn and a metalloid arsenic (As) (Teng et al. 2010).

Besides contamination, urban land expansion also largely encroached upon arable land resources in the last decade. For example, one study has shown that the urban land area in the Beijing–Tianjing–Hebei region (China) expanded by 71% during a 10-year period (1990–2000) (Tan et al. 2005). This problem—the decrease in arable land resource with urbanization and an increase in population—has attracted worldwide attention (Cai et al. 2002; Fazal 2001; She and Xie 2000; Tania et al. 2001). In 1998, China issued an arable land requisition–subsidy balance policy, which promised to subsidize an equal amount of land to farmers when their arable land is requisitioned for non-agricultural use, such as infrastructures for residential and industrial purposes. The aim of this policy was to preserve land resources for agricultural use. However, due to the lack of an effective and reliable arable land evaluation method, the requisition–subsidy balance policy is proven to be ineffective and the loss and degradation of arable land continues (Chen et al. 2015; Hu et al. 2012). Therefore, it is very important to develop a more effective and reliable method for evaluating arable land resources.

The land capability classification released by the US Department of Agriculture in 1961 laid the foundation for the quantitative analysis of arable land resources (Klingebiel and Montgomery 1961). The current international and national quality evaluation systems of arable land tend to focus more on production capability, land potential, and ecological quality and sustainability (Fu and Bai 2015). However, the implementation practices often only consider one single criterion, such as the soil integrated fertility index (IFI) (Brejda et al. 2000; Mu et al. 2018; Wang et al. 2001). The lack of a comprehensive evaluation standard results in noncomparable evaluation results. A more scientific and applicable tool for assessing arable land resources is needed. A practical assessment of arable land resource requires integrated consideration of key soil properties and their spatial and temporal variations (Alaoui et al. 2018). Currently, most of the existing evaluation systems are based on the provincial level yet there is little on the county or town level, although the relationships of available micronutrients in the soil and influencing factors were scale- and location-dependent (Tan et al. 2005; Zhu et al. 2016). This implies that, in order to improve the quality of arable land, different management practices are needed on a smaller scale level.

This study aims to develop a multi-criteria evaluation system for evaluating arable land area by taking into account both the fertility of land and the soil cleanliness index (i.e., metal contamination) as restriction factors. The evaluation system determines arable land resources via a new arable land area correction method, which could provide an effective and reliable method for the evaluation and management of arable land resources.

## Materials and methods

### Study area and backgrounds

The study area, Chongming District (31.45° to 31.85° N and 121.16° to 121.90° E), is located in the Yangtze River estuary of China. Chongming District includes three islands (Chongming, Changxing, and Hengshan), which possess the largest and most concentrated agricultural land resources as well as the best agricultural environment in Shanghai, China. Since almost half of the area of the present islands is from the reclamation of wetland (Zheng et al. 2016), the quality of the reclaimed soil has been a concern, especially due to contamination by heavy metals and metalloids (Yang et al. 2013; Zheng et al. 2016). With the increasing emphasis on arable land quality and management, a series of studies have been carried out on heavy metals in the soils of Chongming District (Hu et al. 2013; Ma et al. 2015; Sun et al. 2010). Extensive agricultural activity has increased the accumulation of heavy metals (e.g., Cr, Zn, Cu, and Zn and As) in paddy fields and farmland (Zheng et al. 2016). In addition, stubble burning is also regarded as a significant source of heavy metals through atmospheric deposition (Sun et al. 2010). Thus, a multitude of factors might possibly affects the arable land assessment of Chongming District, China.

### Sample collection

**Fig. 1:**
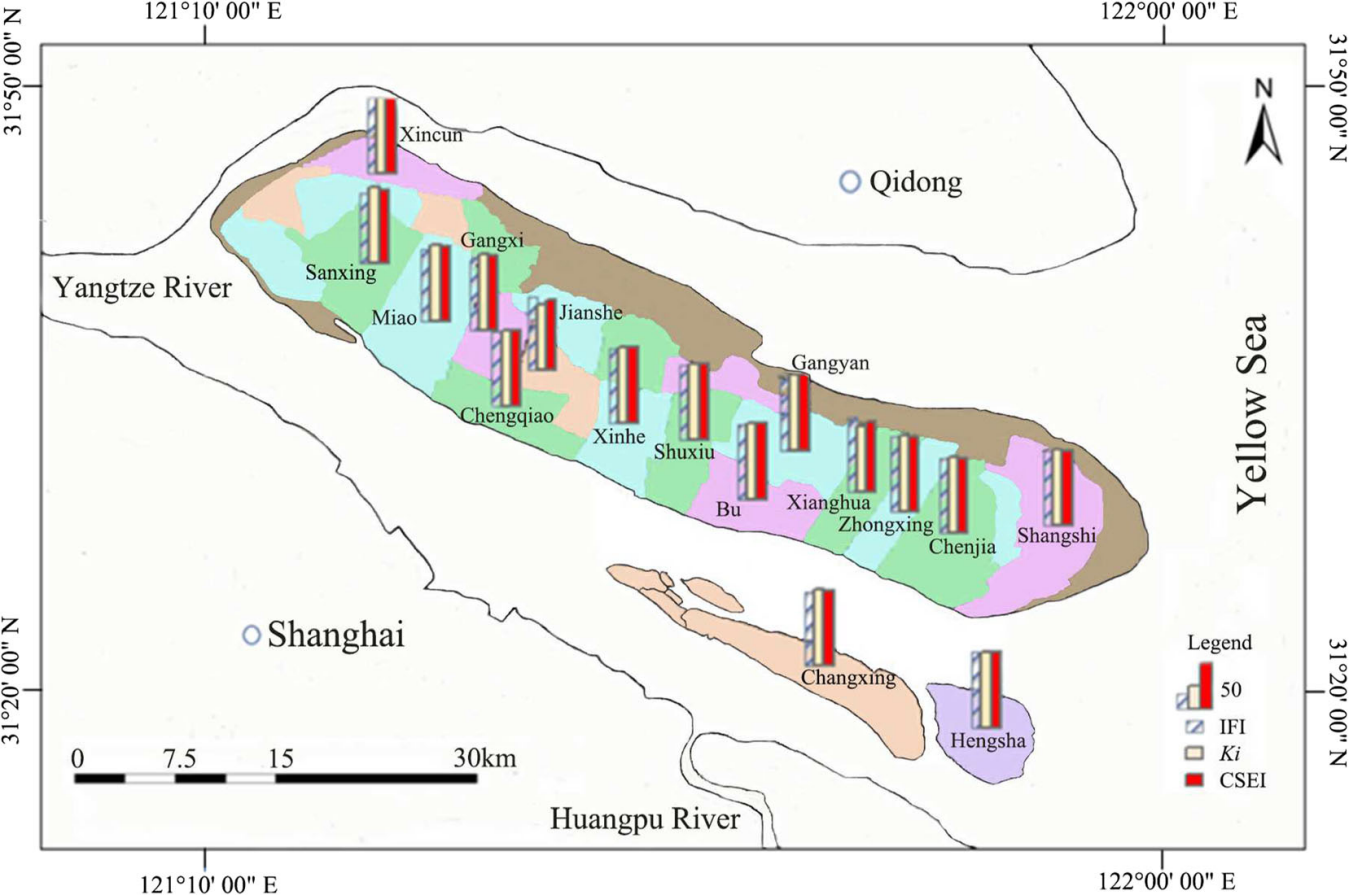
The locations of 16 towns (separated with different colors) sampled in Chongming District, China. Their corresponding soil integrated fertility index (IFI), soil cleanliness index (*K*_*i*_), and comprehensive soil evaluation index scores (CSEI) are shown as columns

Topsoil samples (2 cm to 20 cm) were collected with a bamboo spade in 16 towns of Chongming District in April and July 2016. Each town featured 4 to 7 sampling sites including paddy and upland fields. A total of 104 samples were collected. Figure 1 shows the location of the 16 towns studied in Chongming District. At each sampling site, a 1 × 1 km^2^ sampling grid was randomly selected. Five topsoil cores were collected from each sampling grid, including one central point and four additional points towards the east, west, south, and west. After collection, these five topsoil samples were mixed together to make a single composite sample.

### Sample analyses

After transport to the laboratory, soil samples were oven-dried at 60 °C, ground, and passed through a 75-μm (equivalent to no. 200 according to ASTM E11 standards) stainless steel sieve. Soil samples were stored in a desiccator prior to further analyses. Total organic matter (TOM) was estimated by the potassium dichromate (K_2_Cr_2_O_7_) volumetric method (NY/T 1121.6; Ministry of Agriculture 2006) using the K_2_Cr_2_O_7_–sulfuric acid solution as the digestion medium. Available phosphorus (Av-P) was extracted by sodium bicarbonate and determined by the molybdenum–antimony colorimetric method (NY/T 1121.7; Ministry of Agriculture 2014). Available potassium (Av-K) was extracted by ammonium acetate and measured by flame atomic absorption spectrophotometry (NY/T 889; Ministry of Agriculture 2004). The land fertility levels for TOM, Av-P, and Av-K were assessed based on the classification of soil nutrition adopted by the Second National Soil Survey (National Soil Survey Office 1979).

The concentrations of As, Cu, Cr, Pb, and Zn were measured using inductively coupled plasma mass spectrometry (ICP-MS) (PE NexlON 300X, PerkinElmer). Prior to ICP-MS analysis, 0.1 g soil samples were digested by 3 mL HNO_3_ (65%), 1 mL HF (40%), and 1 mL H_2_O_2_ (30%) in sealed Teflon vessels in a microwave (PreeKem, TOPEX). After transfer to a volumetric flask, HClO_4_ (1 mL) was added to the clear digest to remove the remaining HF. All the acid used in the digestion step was ultrapure and could be used for trace metal analysis. Analytical quality was controlled by using sample replicates, reagent blanks, and an internal standard. The relative standard deviation (RSD) between duplicates was 0.2% to 15.8%. Internal standard solutions including Sc, Ge, In, and Bi were used for ICP-MS analysis to correct the signal bias and drifts caused by the matrix interference. The study did not consider mercury (Hg) and cadmium (Cd), which had concentrations below the detection limit of ICP-MS.

### The assessment of the multi-criteria evaluation system

#### IFI

The assessment of soil fertility is a useful system that helps to improve sustainable land use management. IFI is an effective and important indicator for assessing the quality and degradation of arable land (Mu et al. 2018; Shang et al. 2014). In this study, we calculated the integrated IFI based on TOM, Av-P, and Av-K parameters using a weighted function

1 $$ \mathrm{IFI}=100\sum {F}_i\times {C}_i\ \left(i=1,2,3,\dots n\right) $$

where *F*_*i*_ is the score of the *i*th parameter, which is used to assess soil fertility index, and *Ci* is the weight coefficient of the *i*th parameter of soil fertility. The weight coefficients for TOM, Av-P, and Av-K were 0.600, 0.200, and 0.200, respectively. In order to minimize the effect of temporary fertilization in the evaluation operation, the weight coefficient of TOM in the matrix was one level higher than that of Av-P and Av-K (Jiao et al. 2014; Lee et al. 2004). The score (*F*_*i*_ value) of each measured soil fertility parameter was calculated by its measured absolute value and a standard scoring function (SSF) (Hussain 1997; Shang et al. 2014). An S-pattern function (Eq. (2)) was used to calculate the SSF values of each parameter (Tian and Xin 2006)

2 $$ {F}_i=\left\{\begin{array}{c}0\kern1.50em {u}_i\le {u}_t\\ {}\raisebox{1ex}{$1$}\!\left/ \!\raisebox{-1ex}{$\left(1+{a}_i{\left({u}_i-{c}_i\right)}^2\right)\kern0.40em {u}_t<{u}_i<{c}_i\left(i=1,2,\dots, m\right)$}\right.\\ {}1\kern1.45em {u}_i\ge {c}_i\end{array}\right. $$

where *u*_*i*_ is the measured concentration of soil samples, *c*_*i*_ is the standard index, *a*_*i*_ is a constant, and *u*_*t*_ is the bottom limit of the index. The values of *a*, *c*, and *u*_*t*_ were derived from expert assessments and analysis by Statistical Package for the Social Sciences (IBM SPSS Statistics 24; Li 2012). For TOM, the values of *a*, *c*, and *u*_*t*_ are 0.040, 14.4, and 2.00, respectively. For Av-P, the values of *a*, *c*, and *u*_*t*_ are 0.019, 20.3, and 3.00, respectively. For Av-K, the values of *a*, *c*, and *u*_*t*_ are 0.0007, 138, and 20.0, respectively.

#### Soil cleanliness index (*K*)

The soil cleanliness index (*K*) was optimized by the coefficient construction method of soil environment quality proposed by Lu et al. (2011). The *K* index was evaluated based on a pollution-level determination method

3 $$ \left\{\ \begin{array}{c}{K}_i=100\ \kern3.3pc {P}_{\mathrm{C}}<0.700\\ {}{K}_i=100\frac{\ 3-{P}_{\mathrm{C}\mathrm{i}}}{3-0.7}\kern5pt 0.700\le {P}_{\mathrm{C}}<3.00\\ {}\ \\ {}{K}_i=0\ \kern3.3pc {P}_{\mathrm{C}}\ge 3.00\end{array}\right. $$

where *K*_*i*_ is the cleanliness index in the *i*th unit and *P*_C*i*_ is the comprehensive soil pollution index in the *i*th unit. The cleanliness index in a given area was calculated by the average value of cleanliness index of all sample sites in the area. The comprehensive pollution index (*P*_C_) is defined as

4 $$ {P}_{\mathrm{C}}=\sqrt{\raisebox{1ex}{$\left({P_{\mathrm{A}}}^2+{P_{\mathrm{max}}}^2\right)$}\!\left/ \!\raisebox{-1ex}{$2$}\right.} $$

where *P*_A_ is the mean value of individual pollution indices and *P*_max_ is the maximum value of the individual pollution index. The standards for the levels of pollution were defined as follows: *P*_C_ ≤ 0.700, very clean; 0.700 < *P*_C_ ≤ 1.00, clean; 1.00 < *P*_C_ ≤ 2.00, light pollution; 2.00 < *P*_C_ ≤ 3.00, medium pollution; and *P*_C_ > 3.00, heavy pollution. The individual soil pollution index (*P*_*i*_) is calculated as

5 $$ {P}_i={M}_i/{S}_i $$

where *P*_*i*_ is the individual pollution index, *M*_*i*_ is the measured value of pollution, and *S*_*i*_ is the lower limit of the pollution index, which is based on the China agricultural soil standard (GB15618-1995). The expression *P*_*i*_ ≤ 1.00 refers to a qualified individual pollution index that does not exceed the standard limit. The expression *P*_*i*_ > 1.00 refers to an unqualified individual pollution index that exceeds the standard limit. Both the individual and comprehensive soil pollution indices were calculated according to the technical specifications for the survey and quality evaluation of arable land (NY/T1634-2008) and the Nemerow index method (Kowalska et al. 2016).

#### Comprehensive soil evaluation index

The arable land resource value was calculated using a comprehensive soil evaluation index (CSEI), which is defined as

6 $$ \mathrm{CSEI}=\frac{\mathrm{IFI}+K}{2} $$

In this study, a CSEI of > 60 is considered acceptable, 70–80 is good, and > 90 is excellent.

#### Land area correction method

An “ideal hectare” is defined as a hectare of arable land with a CSEI score of 100. Based on the concept of an ideal hectare, the corrected land area is determined as

7 $$ {S}_{\mathrm{T}}=\mathrm{CSEI}\times S/100 $$

where *S*_T_ is the value of land area after correction and *S* is the measured geometric area of a given area of arable land.

## Results

### Soil fertility and metal content

The concentrations of TOM of all 16 towns were in the range of 13.3 ± 2.61 g/kg to 22.9 ± 3.45 g/kg (Table 1). According to the Second National Soil Survey, 14 of 16 sampled towns were at TOM level 4 (10 g/kg to 20 g/kg; National Soil Survey Office 1979). The TOM concentration in the towns of Miao (21.7 ± 4.44 g/kg) and Chengqiao (22.9 ± 3.45 g/kg) was higher than 20 g/kg, which belongs to level 3 according to the Second National Soil Survey (Table 1). The range of Av-P content in 16 towns was 159 ± 72 mg/kg to 896 ± 196 mg/kg (Table 1). According to the soil agrochemical standards, Av-P content in all 16 towns was at level 1 (> 40.0 mg/kg, Table 1; Nanjing Agriculture University 1996). For Av-K, 8 of 16 towns were at level 1 (> 200 mg/kg), 6 of 16 towns were at level 2 (150–200 mg/kg), and 2 of 16 towns were at level 3 (100–150 mg/kg, Table 1). In general, due to low TOM content, land fertility was at level 4 in 14 of 16 selected towns (Table 1).

**Table 1:** The concentrations (mean ± standard deviation) of total organic matter (TOM, g/kg), available phosphorus (Av-P, mg/kg), and available potassium (Av-K, mg/kg) in soils of 16 towns in Chongming District, China (*n* = 3–7)

Towns	TOM	TOM level	Av-P	Av-P level	Av-K	Av-K level	Soil grade
Xincun	18.4 ± 1.21	10–20	292 ± 89.2	> 40	270 ± 140	> 200	4
Sanxing	18.7 ± 3.91	10–20	175 ± 35.1	> 40	177 ± 78.6	150–200	4
Miao	21.7 ± 4.44	20–30	178 ± 66.4	> 40	114 ± 31.5	100–150	3
Gangxi	18.9 ± 4.42	10–20	159 ± 72.0	> 40	176 ± 68.4	150–200	4
Chengqiao	22.9 ± 3.45	20–30	188 ± 83.4	> 40	151 ± 48.7	150–200	3
Jianshe	18.6 ± 3.24	10–20	177 ± 69.0	> 40	271 ± 153	> 200	4
Xinhe	14.4 ± 2.26	10–20	207 ± 123	> 40	331 ± 160	> 200	4
Shuxin	14.9 ± 3.48	10–20	243 ± 200	> 40	269 ± 129	> 200	4
Bu	14.3 ± 1.87	10–20	288 ± 225	> 40	288 ± 152	> 200	4
Gangyan	13.9 ± 3.17	10–20	374 ± 308	> 40	324 ± 128	> 200	4
Xianghua	15.1 ± 3.39	10–20	367 ± 84.6	> 40	245 ± 181	> 200	4
Zhongxing	13.3 ± 2.61	10–20	896 ± 196	> 40	259 ± 171	> 200	4
Chenjia	13.8 ± 1.57	10–20	407 ± 240	> 40	158 ± 69.7	150–200	4
Hengsha	17.1 ± 2.44	10–20	290 ± 242	> 40	189 ± 62.8	150–200	4
Changxing	15.6 ± 1.16	10–20	221 ± 120	> 40	121 ± 39.2	100–150	4
Shangshi	15.2 ± 2.37	10–20	295 ± 114	> 40	159 ± 51	150–200	4

Concerning metals and As concentrations, the mean concentrations of Cr, Cu, Zn, and As were 64.5 mg/kg, 31.9 mg/kg, 86.0 mg/kg, and 11.7 mg/kg, respectively, which are higher than their corresponding mean background values in China of 61.0 mg/kg, 22.6 mg/kg, 74.2 mg/kg, and 11.2 mg/kg, respectively (Table 2; Chen et al. 2015). According to Chinese soil guidelines, the concentrations of Cr, Cu, Zn, and As belong to level 1 (Table 2). The mean concentration of Pb (mean = 22.5 mg/kg) was generally low across the whole island. Individual samples were found to have relatively high metal contents in a few towns when compared with the background concentration of China (CNEMC 1990). For example, Cu was found to be 39.5 ± 4.97 mg/kg in the town of Chengqiao (Table S1); the concentration of As in Jianshe reached 21.6 ± 3.00 mg/kg (Table S1); the concentrations of Cu and Zn in Shuxin were 37.2 ± 13.3 mg/kg and 111 ± 38.4 mg/kg, respectively (Table S1).

**Table 2:** Statistics of heavy metals and As concentrations (mg/kg) in the soils of 16 towns in Chongming District, China (*n* = 104)

	Cr	Cu	Zn	As	Pb
Mean	64.5	31.9	86.0	11.7	22.5
Maximum	189	56.2	179	20.0	69.3
Minimum	39.5	14.4	49.0	0	14.0
25th percentile	52.4	22.1	71.6	3.14	21.2
50th percentile	56.6	25.7	81.7	7.68	23.8
75th percentile	61.7	31.3	93.7	10.5	26.7
Mean backgrounds in China*	61.0	22.6	74.2	11.2	26.0
Chinese soil guidelines (level 1)	90.0	35.0	100	15.0	35.0
Chinese soil guidelines (level 2)	200	200	250	30.0	300

*The background values in China were obtained from China National Environmental Monitoring Center (CNEMC, 1990). The Chinese soil quality categories are defined according to the report by Chinese Environmental Protection Administration (CEPA, 1995)

### Comprehensive soil evaluation indices

Only 2 (Sanxing and Changxing) of 16 towns had IFI values lower than 95.0. All of the other fourteen towns had high IFI values in the range of 95.3 to 100 (Fig. 1). The IFI values for Sanxing and Changxing were 93.7 and 94.7, respectively. By comparing the scores of TOM (*F*_TOM_), Av-P (*F*_Av-P_), and Av-K (*F*_Av-K_), we found that only Av-K showed lower score values with a range of 0.685 to 1.00 (mean of 0.858). For example, the lowest IFI (93.7) was found in Sanxing, with scores of 1 (*F*_TOM_), 0.685 (*F*_Av-P_), and 1 (*F*_Av-K_), respectively.

**Fig. 2:**
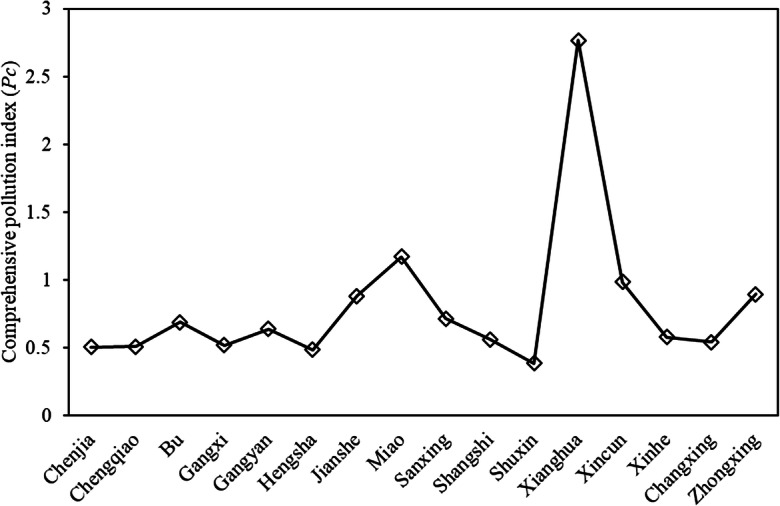
Comprehensive pollution index (*P*_C_) of soils in 16 towns of Chongming District, China

In general, the soil cleanliness indices (*K*_*i*_) of all 16 towns were relatively good, with a range of 84.6 to 100 (mean of 95.3, Fig. 1). The lowest *K* value (84.6; Fig. 1) was found in the town of Xianghua with a highest comprehensive pollution index (*P*_C_) of 2.77 (Fig. 2). There were 10 of 16 towns with *P*_C_ values lower than 0.7 (Fig. 2), suggesting the soils in these towns were at the very clean level based on metal and metalloid contamination. The *P*_C_ values in 4 of 16 towns (Sanxing, Jianshe, Zhongxing, and Xincun) were at the clean level, with a range of 0.72 to 0.99 (Fig. 2). The towns of Miao and Xianghua had high *P*_C_ values of 1.17 and 2.77, respectively (Fig. 2). The soil in Miao was at the light pollution level with a *P*_C_ value of 1.17. Xianghua was at the medium pollution level, with a *P*_C_ value of 2.77 (Fig. 2).

The means of CSEI (Eq. (6)) of 16 towns were in the range of 90.7 to 99.2 with a mean of 96.2, suggesting that the soils in all of the towns were at the excellent level (Fig. 1). The lowest CSEI value of 90.7 was found in the town of Changxing due to its low IFI (94.7) and *K* (86.6) values (Fig. 1). The order of towns based on CSEI value was completely different than the orders based solely on the IFI or soil cleanliness index.

### Land area correction

**Fig. 3:**
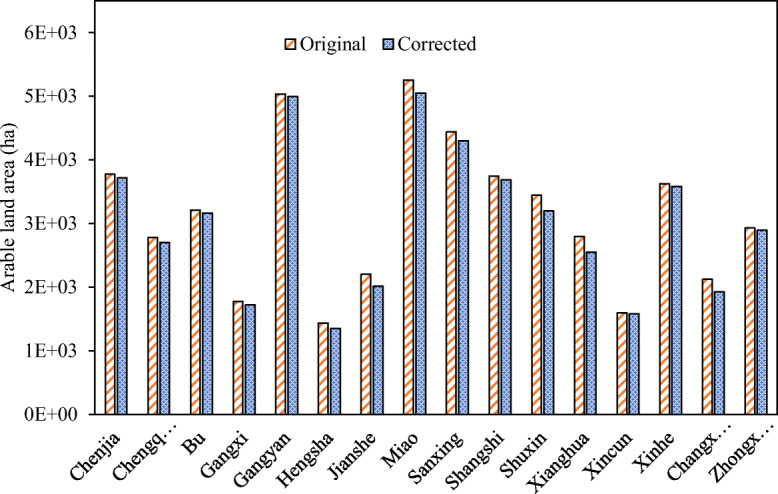
Arable land area correction results for 16 towns in Chongming District, China. “Original” is the measured geometric area, and “Corrected” is the corrected land area after CSEI correction

The arable land in the town of Gangyan, with a CSEI value of 99.2, was close to the so-called ideal hectare. A relatively lower corrected area of arable land, in comparison to their measured geometric area, was found in the towns of Hengsha, Shuxin, Jianshe, Xianghua, and Changxing (Fig. 3). For example, in Changxing, the corrected arable area accounted for only 90.6% of its original measured arable area because of its relatively low fertility and cleanliness indices (Figs. 1 and 3).

## Discussions

A number of studies have reported the soil quality of arable land in Chongming District, mainly focusing on the distribution and quality assessment of heavy metals and dissolved OM (Lou et al. 2017; Sun et al. 2010; Wang et al. 2015; Zheng et al. 2016). The previous studies together with the results obtained in the present study show that the soil quality of the agricultural land in Chongming District is generally good (Zhang et al. 2014; Zheng et al. 2016; this study). However, sustained attention and management is still necessary due to the potential risk of heavy metal accumulation and soil degradation problems (Zhang et al. 2014; Table 2 in this study).

Due to rapid economic development, soil pollution by heavy metals has been widespread in China since the late 1970s (Chen et al. 1999). In general, in this study, we found that the heavy metal levels in Chongming District were good, with most of heavy metals (based on mean values of each town) belonging to the level 1 category of Chinese soil guidelines (CEPA 1995). However, with large variations, the mean concentrations (*n* = 104) of Cr, Cu, Zn, and As were higher than those of the mean backgrounds in China (see Tables 2 and 3). Moreover, the mean concentrations of Cr, Cu, Zn, and As found in the present study were relatively higher than those taken in the previous studies within the same area (Wang et al. 2007; Zheng et al. 2016; Table 3). These results indicate that there is a possibility that the concentration of heavy metals and As has accumulated in recent years. The over-application of pesticides and stubble burning may partly explain the accumulation of heavy metals in agricultural soils in Chongming District (Sun et al. 2010).

**Table 3:** Ranges of concentrations of Cr, Cu, Zn, As, and Pb in this study and values found in previous studies

Sampling site (agriculture soils)	Cr	Cu	Zn	As	Pb
This study	43.8–189 (78.9 ± 6.58)	20.6–39.5 (31.9 ± 7.56)	67.8–111 (86.1 ± 19.5)	0–21.6 (11.7 ± 5.0)	10.8–34.4 (22.5 ± 7.66)
Kermanshah, Iran (Doabi et al. 2019)	32.0–235 (133.5 ± 101.5)	10.0–83.0 (46.5 ± 36.5)	40.0–113 (76.5 ± 36.5)	ND	ND
Pakhtunkhwa, Pakistan (Khan et al. 2013)	0.29–0.64 (0.47 ± 0.18)	0.28–0.61 (0.45 ± 0.17)	0.20–0.52 (0.36 ± 0.16)	ND	ND
Telangana, India (Adimalla et al. 2019)	55.9–135.8 (95.9 ± 40.0)	12.7–69.6 (41.2 ± 28.5)	71.3–173 (122 ± 50.9)	2.40–5.3 (3.85 ± 1.45)	5.90–26.8 (16.4 ± 10.5)
Morocco (Oumenskou et al. 2018)	16.1–294 (155 ± 139)	1.46–191 (96.3 ± 95)	24.5–1272 (648 ± 624)	ND	3.40–135 (69 ± 66)
Colombia, America (Marrugo-Negrete et al. 2017)	0.01–0.08 (0.045 ± 0.035)	12.6–2522 (1267 ± 1257)	285–2632 (1459 ± 1174)	ND	0.02–0.13 (0.075 ± 0.055)
Odo-Oba, Nigeria (Adagunodo et al. 2018)	23.0–341 (182 ± 159)	3.91–20.7 (12.3 ± 8.39)	22.8–61.3 (42.1 ± 19.3)	1.60–3.70 (2.65 ± 1.05)	19.0–43.9 (31.4 ± 12.5)
Serbia (Saljnikov et al. 2019)	25.6–100 (62.6 ± 37.0)	20.4–109 (64.8 ± 44.4)	50.7–125 (87.9 ± 37.2)	4.89–54.1 (29.5 ± 24.6)	4.77–171 (88.1 ± 83.3)
Guangdong, China (Cai et al. 2019)	5.70–57.1 (31.4 ± 25.7)	1.20–48.6 (24.9 ± 23.7)	25.1–106 (65.6 ± 40.5)	1.80–25 (13.4 ± 11.6)	25.6–84.9 (55.3 ± 29.7)
Sihui, Guangdong, China (Zhang et al. 2018)	ND	4.60–62.3 (33.5 ± 28.9)	ND	3.31–83.1 (43.2 ± 39.9)	13.3–71.3 (42.3 ± 29)
Taiyuan, China (Liu et al. 2015)	14.6–193 (104 ± 89)	5.83–274 (140 ± 134)	169–278.6 (148 ± 131)	0.62–23.5 (12.1 ± 11.4)	6.32–73.7 (40.0 ± 33.7)

Values in the brackets are the mean ± standard deviations

*ND* not detected

As the quality of arable soil changes with agricultural practices and anthropogenic activities, the evaluation system for arable land resources also needs development and renewal with time. The multi-criteria evaluation system integrating the IFI and the soil cleanliness index (*K*) proposed in this study provides a new evaluation method for the arable land resources. When the proposed system was applied to evaluate arable land based on town unit in Chongming District, the CSEI values of the 16 towns were found to range from 90.6 to 99.2 (Fig. 1). The town of Changxing, which had the lowest CSEI value (90.6), was also found to have a low *K* value of 86.6 (Fig. 1). In this study, in order to calculate the IFI, three parameters (TOM, Av-P, and Av-K) were selected, of which Av-P and Av-K were indicators of nutrient status and TOM influenced the biological activities in the soil habitat. Besides the IFI, the introduction of the soil cleanliness index made the comprehensive evaluation of arable land more reliable. The order of the 16 towns based on the multi-criteria evaluation system (CSEI value) was different from the orders based solely on the IFI or soil cleanliness index, indicating the proposed multi-criteria evaluation system provides a better assessment for arable land area correction.

A practical and reliable system for evaluating arable land resources requires the integrated consideration of key soil properties (e.g., fertility and contamination indicators) and their variations across space and time. However, the current arable land evaluation system does not cover all of these aspects (Cui et al. 2010; Wu et al. 2011). Productivity and quality indicators (e.g., fertility parameters) are currently the main parameters that can be used for land evaluation systems (Hao et al. 2015; Liu et al. 2014; Wang et al. 2014). The complex data acquisition process is another hurdle that exists in the poor implementation of current evaluation systems for arable land resource value (Duru et al. 2010; Torbert et al. 2009). For example, in many evaluation systems, data acquisition and processing often require large-scale instrumentation monitoring systems and large sample sizes, which local governments view as significant obstacles. Therefore, the present study’s emphasis on arable land ecology on a small regional scale, combining both the IFI and the soil cleanliness index, has made it feasible to evaluate land resources related to both land fertility and ecological quality.

Returning to the requisition–subsidy balance policy, farmers are the most basic interest group for the nonmarket value of arable land resources, so their needs should be given special attention. In the practice of arable land requisition and subsidy, the subsidy amount can be calculated based on the corrected land area and a predefined price for the ideal hectare. In other words, the subsidy can correspond to the ideal hectare of arable land. This type of subsidy can better reflect the quality of arable land and soils, encouraging the authorities and farmers to pay more attention to the protection of arable land and guide a more reasonable and reliable subsidy policy for arable land in the future. In the end, this multi-criteria evaluation system may also provide an effective assessment tool for the management of administrative organization and assist in the improvement of arable land quality and healthy agricultural products.

## Conclusion

This study developed a multi-criteria evaluation system by combining IFI with the soil cleanliness index as two restriction factors. The calculated comprehensive soil indices of the 16 towns in Chongming District ranged from 90.6 to 99.2 with a mean of 96.2. All these arable lands fell into the excellent category. This new multi-criteria evaluation system of arable land resources better reflects the soil fertility and pollution status. In the future, this multi-criteria evaluation system can be used with more integrated fertility parameters and soil cleanliness indices depending on the properties of the arable land.

The proposed multi-criteria system also provides a new direction and method for the evaluation of arable land as well as for its sustainable use. The evaluation system determined arable land resource values via the land area correction method. By linking the concept of the ideal hectare to subsidy amount, an equivalent ideal hectare of arable land can be determined based on the predefined price of an ideal hectare and land area correction results. This new type of subsidy can help the agricultural authorities and farmers focus on the protection and quality of arable land as well as create a reasonable subsidy policy for arable land. In conclusion, this study provides an easy and effective method to measure arable soil quality and potentially guarantee the quality of agricultural products.

## Electronic supplementary material

ESM 1 (DOCX 15 kb)
