# Supplementary material for: A multi-criteria evaluation system for arable land resource assessment
Source: Environ Monit Assess. 2020 Jan 2;192(2):79. doi: 10.1007/s10661-019-8023-x (PMC6940353; doi:10.1007/s10661-019-8023-x)
Supplement: Supplementary file 1 — (DOCX 15 kb) [file 10661_2019_8023_MOESM1_ESM.docx]

**Supplementary Information**

**A multi-criteria evaluation system for arable land resource assessment**

Feipeng Li^1^, Wei Liu^1^, Zhibo Lu^2^, Lingchen Mao^1^, Yihua Xiao^3,4*^

^1^School of Environment and Architecture, University of Shanghai for Science and Technology, Shanghai 200093, China

^2^College of Environmental Science and Engineering, Tongji University, Shanghai 200092, China

^3^Department of Biological and Environmental Science, University of Jyväskylä, Jyväskylä 40014, Finland

^4^School of Environmental & Municipal Engineering, Qingdao University of Technology, Qingdao 266033, China

*Corresponding Author: Yihua Xiao

Email: [yihua.y.xiao@jyu.fi](mailto:yihua.y.xiao@jyu.fi)

Telephone: +358 458601970

ORCID: 0000-0001-8643-4421

Table S1. The concentrations (mean ± standard deviation, mg/kg) of heavy metals and arsenic (As) and their levels in soil of 16 towns in the Chongming district, China (*n* = 3–7)

| Towns | Cr | Cu | Zn | As | Pb | Soil grade |
| --- | --- | --- | --- | --- | --- | --- |
| Xincun | 54.0 ± 4.66 | 22.8 ± 3.40 | 71.0 ± 5.05 | 0.0 | 14.3 ± 1.52 | One |
| Sanxing | 55.0 ± 3.86 | 20.6 ± 1.15 | 67.8 ± 13.2 | 2.63 ± 1.36 | 10.8 ±3.04 | One |
| Miao | 43.8 ± 2.75 | 27.5 ± 4.85 | 71.5 ± 15.3 | 7.81 ± 4.66 | 12.5 ±1.22 | One |
| Gangxi | 62.5 ± 1.64 | 28.5 ± 2.24 | 73.3 ± 4.18 | 7.83 ± 4.07 | 22.0 ± 1.38 | One |
| Chengqiao | 58.5 ± 0.84 | 39.5 ± 4.97 | 90.3 ± 7.94 | 4.38 ± 1.48 | 22.5 ± 0.73 | Two |
| Jianshe | 68.1 ± 4.63 | 28.0 ± 4.92 | 74.0 ± 9.38 | 21.6 ± 3.00 | 18.1 ±6.85 | Three |
| Xinhe | 64.1 ± 5.12 | 31.2 ± 10.9 | 99.9 ± 23.3 | 15.0 ± 4.05 | 24.6 ± 3.31 | One |
| Shuxin | 68.4 ± 9.95 | 37.2 ± 13.3 | 111 ± 38.4 | 11.8 ± 7.16 | 29.7 ± 10.1 | Two |
| Bu | 61.6 ± 11.1 | 29.6 ± 6.15 | 86.1 ± 12.6 | 14.7 ± 5.65 | 26.4 ± 2.10 | One |
| Gangyan | 60.3 ± 8.44 | 30.8 ± 8.81 | 82.4 ± 19.6 | 13.7 ± 3.79 | 26.6 ± 12.7 | One |
| Xianghua | 92.8 ± 23.5 | 33.8 ± 6.06 | 86.9 ± 18.7 | 11.3 ± 2.49 | 26.7 ± 3.92 | Two |
| Zhongxing | 73.7 ± 6.03 | 35.7 ± 4.07 | 94.9 ± 8.46 | 12.5 ± 4.40 | 23.9 ± 2.81 | Two |
| Chenjia | 68.3 ± 3.94 | 37.3 ± 3.64 | 97.1 ± 13.5 | 17.0 ± 2.40 | 24.4 ± 3.76 | Two |
| Hengsha | 62.5 ± 8.29 | 34.4 ± 4.29 | 93.9 ± 15.9 | 13.0 ± 4.23 | 34.4 ± 16.0 | One |
| Changxing | 72.3 ± 7.63 | 35.5 ± 3.97 | 88.6 ± 13.4 | 16.6 ± 4.20 | 22.7 ± 9.83 | Two |
| Shangshi | 66.4 ± 8.74 | 37.7 ± 6.50 | 88.3 ± 15.3 | 17.4 ± 2.49 | 20.4 ± 4.16 | Two |
